# Supplementary material for: Two Cases of Pancytopenia with Coombs-Negative Hemolytic Anemia after Chimeric Antigen Receptor T-Cell Therapy
Source: Int J Mol Sci. 2021 May 21;22(11):5449. doi: 10.3390/ijms22115449 (PMC8196726; doi:10.3390/ijms22115449)
Supplement: Supplementary file 1 [file ijms-22-05449-s001.zip › ijms-1196566-supplementary.pdf]

Supplementary material

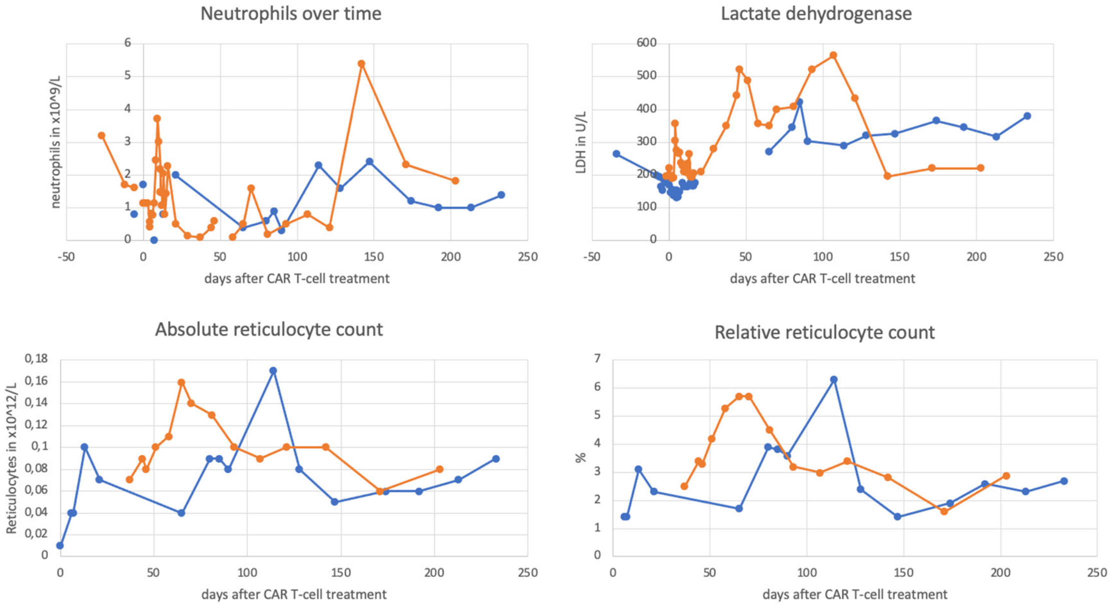

<u>Supplemtary Figure S1: Blood cell counts over time</u>

Blue = patient A, orange = patient B

Supplementary figure 2

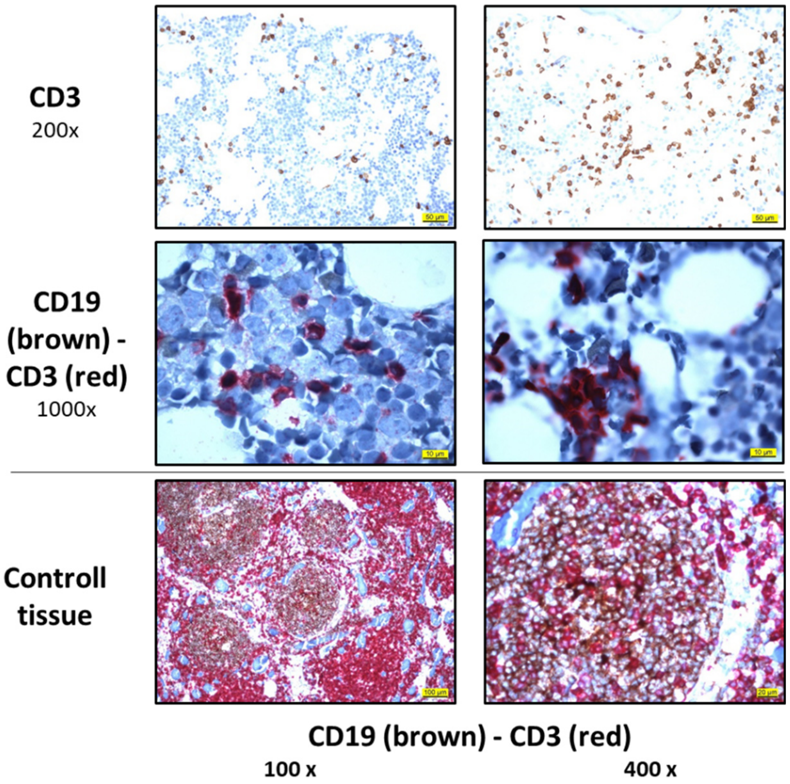

Supplementary Figure S2: Immunohistochemical staining of CD3 and CD19. The immunhistochemial stainings of CD3 revealed a low to moderate increase of T-cells in the interstitium of the bone marrow of both patients, whereby a double-staining of CD19 (brown) and CD3 (red) did not show any overlay. A control tissue shows the typical distribution of CD19 positive B-cells in the follicular and of CD3 positive T-cells in the interfollicular area of a reactive lymph node. Finally, CD20 and CD79a detected no abnormal B-cell [illegible]

Supplementary Table S1: Lab values over time for patient A

Days after CAR T-cell treatment	Hemoglobin (g/dL)	absolute reticulocytes ($x10^{12}/L$)	relative reticulocytes (%)	haptoglobin (mg/dL)	LDH (U/L)	Thrombocytes ($x10^9/L$)	Leukocytes ($x10^9/L$)	Neutrophiles ($x10^9/L$)
-34	10,6				264	98	5,2	
-8	9,8				199	60	2,33	
-8	9,7					67	2,44	
-6	9				194	52	2,5	0,8
-6	8,7					60	2,38	
-5	8,7				164	59	2,32	
-4	7,9				154	47	1,84	
-3	10,4				185	50	2,56	
-2	10,6				185	49	2,71	
-1	10,5				177	48	2,28	
0	10,2	0,01	0,4		170	45	1,93	1,7
0	10,7					48	1,83	
1	9,4				146	37	2,26	
2	9,8				155	33	3,39	
2	9,8					34	2,6	
3	9,2				137	28	1,31	
3	9,5					27	0,85	
4	9,1				139	24	0,29	
4	8,5					23	0,27	
5	8,4				130	20	0,27	
5	7,9					20	0,31	
5	8,1				153	19	0,3	
6	8,6					21	0,33	
6	8,5	0,04	1,4		133	20	0,33	
6	7,6					20	0,39	
6	7,7					21	0,55	
7	8,7					23	0,66	
7	8,8	0,04	1,4		150	24	0,64	0
7	7,4					22	0,57	
8	8					25	0,69	
8	9,4					27	0,86	
9	11				175	36	1,44	
10	10,6				164	40	1,4	
11	9,9				165	41	1,61	
12	10				165	44	1,91	
13	10					56	2,09	
13	10,2	0,1	3,1			47	2,14	0,8
14	10,1				177	48	2,28	
15	10,1				166	46	2,49	

16	10,2				168	47	2,15	
17	10,1				176	46	2,06	
21	10,1	0,07	2,3			40	3,55	2
65	8,5	0,04	1,7	0	272	30	1,68	0,4
80	8,6	0,09	3,9	0	345	20	1,79	0,6
85	8,4	0,09	3,8	0	422	15	2,32	0,9
90	8,3	0,08	3,6		303	26	1,44	0,3
114	9,9	0,17	6,3	38	290	32	3,62	2,3
128	11,1	0,08	2,4		321	37	3,03	1,6
147	11,5	0,05	1,4		326	61	4,5	2,4
174	10,9	0,06	1,9	91	365	51	3,05	1,2
192	10,7	0,06	2,6	91	345	57	2,48	1
213	10,3	0,07	2,3		317	61	2,22	1
233	11,4	0,09	2,7		380	78	2,91	1,4

Supplementary Table S2: Lab values over time for patient B

Days after CAR T-cell treatment	Hemoglobin (g/dL)	absolute reticulocytes ($x10^{12}/L$)	relative reticulocytes (%)	haptoglobin (mg/dL)	LDH (U/L)	Thrombocytes ($x10^9/L$)	Leukocytes ($x10^9/L$)	Neutrophiles ($x10^9/L$)
-27	13,6					95	4,36	3,19
-12	13,6					73	2,59	1,7
-6	12,80					79	2,4	1,61
-2	12,60				197	70	2,2	
0	12,80				222	72	1,2	1,14
1	12,00				198	57	1,2	1,15
2	11,30				189	57	1,2	1,14
3	10,40				191	55	1,3	1,14
4	10,60				304	34	0,6	0,58
4	9,60				356	35	0,5	0,41
5	10,20				276	28	0,9	0,83
6	10,00				267	30	1	0,77
7	10,30				268	29	1,8	1,13
8	10,70				237	34	3,9	2,46
9	10,40				228	44	5,8	3,71
10	10,50				211	60	4,6	3,01

11	11,30				224	83	3,6	2,19
11	11,10				212	72	2,5	1,48
12	12,20				206	98	2,5	1,46
12	11,70				235	85	1,3	1,07
13	11,60				264	90	2,7	2,04
14	10,70				195	75	1,3	0,81
15	11,10				194	72	1,8	1,44
16	11,2				205	74	2,7	2,28
21	10,70				209	39	1,3	0,51
29	10,7				280	23	0,7	0,14
37	10,2	0,07	2,5		350	29	0,76	0,1
44	9,1	0,09	3,4	0	442	27	1,17	0,4
46	8,6	0,08	3,3	0	523	24	1,11	0,6
51	8,5	0,1	4,2	0	489	34	1,25	
58	7,7	0,11	5,3	0	357	29	0,67	0,1
65	10	0,16	5,7	0	351	35	1,37	0,5
70	9,3	0,14	5,7	0	401	43	2,07	1,6
81	10,3	0,13	4,5	0	408	43	0,69	0,2
93	11,4	0,1	3,2	0	523	61	0,98	0,5
107	10,8	0,09	3		564	61	1,47	0,8
121	9,9	0,1	3,4	116	433	76	1,19	0,4
142	12,2	0,1	2,8	64	196	96	6,55	5,4
171	13,6	0,06	1,6	46	221	88	3,49	2,32
203	13,6	0,08	2,9	37	221	88	3,05	1,81
